# How Income Influences Health: Decomposition Based on Absolute Income and Relative Income Effects

**DOI:** 10.3390/ijerph182010738

**Published:** 2021-10-13

**Authors:** Xiaodong Cui, Ching-Ter Chang

**Affiliations:** 1 Business School, Nanjing Xiaozhuang University, Nanjing 211171, China; cui_xiaodong@163.com; 2 Department of Information Management, Chang Gung University, Taoyuan 33302, Taiwan; 3 Clinical Trial Center, Chang Gung Memorial Hospital at Linkou, Taoyuan 33305, Taiwan; 4 Department of Industrial Engineering and Management, Ming Chi University of Technology, New Taipei 24301, Taiwan

**Keywords:** the elderly health, absolute income, relative income, accumulative advantage

## Abstract

Previous research has confirmed a positive association between income and health, but there are still a lot of inconsistencies on how income affects health. Indeed, this impact is caused by overlaying of absolute income and relative income effects, and only by decomposing and comparing their relative importance within an integrated framework can suggestions be made for health inequalities and health intervention. To deal with this issue, using the panel data from the 2011, 2014, and 2017 waves of the Chinese Longitudinal Healthy Longevity Survey (CLHLS), a well-designed research model is established to decompose and explore the impact. Our results indicate that relative income, rather than absolute income, has a significant negative impact on health performance, and that these associations may be causal in nature. The health inequity persists throughout the life cycle, but it remains relatively stable, without significant expansion or convergence. To some extent, the research-proposed models enrich the related literature on associations between income and health, and the empirical results suggest that as China moves to the stage of higher incomes and accelerated aging, the Chinese government should pay more attention to income inequality and be alert to the risks of “income-healthy poverty” traps.

## 1. Introduction

Health is considered as one of the fundamental rights of human beings. WHO (1946) proposed the strategic goal of health for all members, which not only refers to a good health level, but also includes health equity among the people. At the same time, health problems are not only individual problems, but also social problems. Dealing with the problem of health stratification and narrowing the health gap between classes is of great significance to alleviating the contradictions between the rich and the poor and promoting social fairness, justice, and harmonious development. Therefore, the Chinese government has always adhered to the principle of health priority, and health equity has been written into the “Healthy China 2030 Plan”.

In a lot of literature on health equity or health disparity, the relationships between income and health have been focused on in the fields of economics, public health, and welfare economics. Starting from different disciplines and theoretical paradigms, researchers have reached the consensus that the rich are relatively healthier. Health economics does not require economic status or individual income to be considered, but horizontal equity (i.e., equal needs deserve equal care). However, in the real world, economic income is still an important factor influencing health. Regardless of objective health indicators including death rate and disability rate or subjective health indicators including self-rated health and cognitive function, the pro-rich characteristic of health has been proven [1,2,3,4,5]. The World Report on Disability (2011) indicates that 80% of people with disabilities (15% of the world’s population) live in low-income and low- and middle-income countries, which also supports this conclusion.

Since income influences health, can we improve health by increasing income? How does income influence health? What is the entry point of the health intervention policy? There are still many inconsistent conclusions with respect to issues of improving health equity in the literature. Among them, the absolute income hypothesis in the health inequality theory and the relative income hypothesis proposed by Robert Easterlin are the most representative. Discussions on different theories from different perspectives may be the reason for inconsistent income health effects. In fact, the influence of income on health is the superposition of two effects. Only by decomposing the two effects and comparing their relative importance in the same framework can we provide a reference for improving health inequality and seeking health intervention approaches. Based on this, this article constructs a decomposition method of the two effects under the same framework and designs ideas on how to control one effect when discussing the other effect. On this basis, it constructs an absolute income and relative income effect test model and further examines the age characteristics of the effect. In view of the increasingly severe health burden arising from China’s large population and ageing development trend, this study is focused on the aged population. The result shows that the increase of absolute income does not necessarily mean the synchronous improvement of elderly health, relative income is the main cause of health disparity, and the health disparity among different income groups persists throughout the life cycle, but is relatively stable, and there is no significant expansion or convergence with age.

## 2. Literature Review

How does income influence health? Previous studies can be roughly classified on this issue into two explanatory paths, the “Absolute Income Hypothesis” and the “Relative Income Hypothesis”. The absolute income hypothesis based on the health inequality theory emphasizes the positive effect of income on health and starts with the external macro-mechanism to explore the influence mechanism. We believe that the increase of the absolute income level means the development of the macroeconomy, which is conducive to improving the supply of common resources concerning education, medical health, and social security, and the improvement of such resources helps to improve the national health level. The hypothesis has been supported by many researchers [6,7,8]. In addition, some researchers have considered the direct influence of incidental effects on individual health while emphasizing the positive effect of macroeconomic development. For example, macroeconomic development may result in environmental health poverty [9,10]; the increase of income causes the increase of working time, working stress, etc.; such incidental effects restrict the improvement of individual health. Thus, the health effect of absolute income may be weakened [11,12]. Some studies also consider that the health effect of absolute income has a characteristic of diminishing marginal utility because of employment-related stresses [13] or difficulties balancing professional and domestic expectations [14]; the health return of income is not monotonous, but shaped like an inverted U; when the income level exceeds the threshold level, its marginal effect will weaken, and health will have little to do with income [15,16,17].

The relative income hypothesis believes that health depends on not only the absolute income level but also the ranking of individuals in the income distribution sequence. From the perspective of microscopic individuals, it deems that rich people have more critical resources (e.g., knowledge, power, prestige, and favorable social relations) to avoid risks and reduce diseases [18,19]. From the perspective of social psychology, it considers that different income groups face different psychological and social pressures; the low-income group faces bigger psychological pressure and a stronger sense of social deprivation and sense of lack of control, which are related to a series of health problems [8,20]. It is true that the health level can be improved by the increase of income, but when the income of all people rises simultaneously, it means that individual income has no increase on a relative level. As a result, individuals will not psychologically feel the stimulus of income growth. Therefore, the increase of the absolute income level does not necessarily mean the improvement of the health level. Actually, when researching a happiness topic, American economist Easterlin found that happiness is independent of income in the long run, and so is health [21]. Later, more empirical studies found that the group of higher socioeconomic status has longer life expectancy and longer health-adjusted life expectancy [22,23]. Furthermore, some economic literature doubts all causal relations from income to health. On the contrary, health is believed to be the result of the social selection mechanism; it is not income that influences health, but health influences people’s gaining of income. Health problems cause downward social mobility, and upward social mobility often has good health conditions [24].

There is a controversy concerning the studies on the relationship between income and health, i.e., the health returns of income show inconsistency in different life periods. The convergence hypothesis believes that the health return of income gradually expands in the initial elderly stage but continuously shrinks in the late elderly stage [10,25]. The interpretation of the convergence hypothesis is with the increase of age, the difference in psychological risk factors faced (e.g., lack of social relations or social support, loss of the sense of control) by different income groups gradually shrinks and even disappears; the determinative effect of biological factors on health gradually increases and even surpasses the role of socioeconomic factors. The empirical studies carried out by Mirowsky and Link et al. support this view [26,27], and they found that before the age of 80, different income groups have a large health disparity; after the age of 80, the disparity gradually decreases. However, some studies have found that for both men and women, the health advantages of income are continuously accumulated throughout the life course as time goes by, causing the health disparity among different socioeconomic status groups to continue to expand rather than shrink [28]. This viewpoint is known as the “Cumulative Advantage Hypothesis”.

The literature above provides a beneficial exploration focusing on a hypothesis of income influencing health, but unfortunately, previous studies focused only on single hypotheses are incapable of comparing the relative importance of two hypotheses in the same research framework, and even the same hypothesis fails to give a clear answer. Thus, it is difficult to provide the appropriate entry point of the health promotion intervention policy. In addition, the existing literature is largely based on the experience of Western developed countries, and it is debatable whether these studies’ conclusions are applicable to China. The latter is largely different from the former in terms of economic development level, social system, and population structure, and the relations between income and health may be in different forms. Therefore, in this study, we attempt to bring absolute income and relative income into the same framework for comparison. In view of the increasingly severe health burden arising from China’s “Large Population and Ageing” development trend, this study is focused on the health of China’s elderly population, and CLHLS data are utilized to investigate the influence of income on health. Specifically, it includes the following questions:

Question 1: In the view of the absolute income theory, the increase of the absolute income level means the development of the macroeconomy, which is conducive to improving the national health level while improving medical security and public services. Hence, the first issue of this study is to confirm whether the increase of the absolute income level improves the health status of the elderly group, to test the sensibilities of the absolute income effect on health in different income groups and to provide Chinese answers to the theory of diminishing marginal effect of absolute income.

Question 2: In the view of the relative income theory, different income groups represent the utilization degree of critical resources and medical services, and the difference in the capabilities to own and use such resources results in health inequality. Thus, the second issue of this study is to confirm the applicability of this conclusion in China and to investigate the health disparity among different income groups. Moreover, the relative income theory holds that with severer income inequality, the low-income group will have a stronger sense of social deprivation, resulting in a bigger health disparity among different income groups. Therefore, this study further verifies whether the hypothesis that a bigger income difference results in a bigger health disparity is true.

Question 3: There are two different views on whether the impact of income on health will change with age. In the view of the convergence hypothesis, the health disparity continuously shrinks in the late elderly stage; in the view of the cumulative advantage hypothesis, the accumulation of income health advantages results in the continuous expansion of the health disparity. Thus, in this study, the manifestation of the health disparity among different income groups, i.e., the characteristic of health disparity varying with age, will be researched.

## 3. Method

### 3.1. Research Design

**Figure 1:**
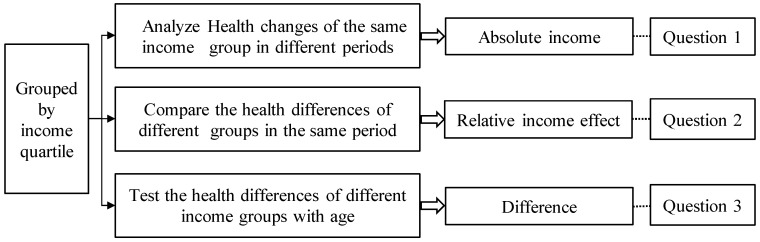
Research framework design.

The key to accurately identifying the two effects of absolute income and relative income and comparing their importance is how to decompose the two effects within the same framework, that is, how to control one effect when discussing the other effect. The research framework design of this study is as shown in Figure 1: first, the samples of each observation period are grouped by quartile of income series into low-income, low- and middle-income, middle- and high-income, and high-income groups. Next, the health changes of the same income group in different periods are analyzed, which can be considered as the absolute income effect, and the absolute income effects of different income groups are compared, which means the sensibility. The two tests correspond to Question 1. Then, the health difference among groups in the same observation period is explored and compared, which means the relative income effect corresponding to Question 2. Finally, the characteristic of health difference with age is tested, which corresponds to Question 3.

Design logic and assumptions: first, the samples are classified into different income groups using the quartile method, wherein, the individuals of the same group are at the equivalent income level and have equivalent critical resources. It can be assumed that there is no relative deprivation effect in the same group, and the health changes after the increase of income can be deemed as the absolute income effect excluding the relative income influence; considering that the average income level of the same group increases accordingly in different observation periods as seen from columns 2 to 4 in Table 1, the absolute income effect is manifested as the health changes of the same group in different periods; second, the relative income effect can be researched by exploring the health disparity among different groups in the same period, because the comparison of different groups in the same period eliminates the influence of macro environmental changes (i.e., absolute income effect) on health level. The hypothetical premise of the relative income effect is a significant income gap between the rich and the poor. During the sample survey period, the Gini coefficient of Chinese residents’ income is greater than the warning line 0.4 of the income gap stipulated by the United Nations Development Program (as shown in column 4 in Table 1), which supports this hypothesis. Finally, it is worth noting that although relative income is divided according to the absolute income quartile, the relative income effect emphasizes the difference in income group rather than the amount of income. Income quantiles are used to represent income levels, because income generally follows power-law distribution and belongs to the statistics of extreme values, and quantile statistics are more representative than traditional mean values.

### 3.2. Model Building

#### 3.2.1. Data and Variables

The data of this study come from the data of the last three surveys (2011, 2014, and 2017) with respect to the CLHLS project of the Center for Ageing and Health, Peking University. The respondents are aged 65 and above. For the CLHLS project, the multistage random unequal proportion sampling method is adopted, with the scope covering 22 provinces/municipalities/autonomous regions nationwide, the number of respondents accounting for 85% of China’s total population. As high-quality survey data, the project has been adopted by lots of institutes. For the detailed survey design of CLHLS, see the website of the Institute of Social Science Survey, Peking University.

The dependent variable in this study is self-rated health status (*HI*). Self-rated health is somewhat subjective but more inclusive and accurate than such indicators as physical function status and disease occurrence rate. It can preferably predict death rate, incidence rate, and so forth and has become one of the most common health survey indicators [29]. The question corresponding to *HI* data is “What do you think of your health status?” and is arranged with five options, “Very Good, Good, Fair, Poor, Very Poor”, to which the points of 1–5 are assigned, respectively. The less the points are, the healthier the respondent will be.

The core explanatory variable is individual income. Individual income means individual absolute income in a year. In the CLHLS project, income refers to gross annual household income, so gross annual household income divided by family size equals individual income (the income in each period has been deflated according to the inflation index) in this study. Other control variables include characteristic variables reflecting individual natural attributes (age, gender, residence), economic conditions (pension, insurance, early life stage, etc.), health habits (smoke or not, drink or not), and the like. Variables are defined as in Table 2.

#### 3.2.2. Test Model

According to the above research issues and design idea, the absolute income test is equivalent to the test of health disparities of the same income sequence in different observation periods, and the relative income test comes down to the test of different income groups in the same observation period. Different observation periods and different income groups can be both deemed as categorical variables. Then, the existence or significance of an effect can be determined by exploring the significance of the categorical variable. Categorical variables are usually added in the form of dummy variables. Thus, the test model of income health effects added with dummy variables is built in this study. With regard to whether the changes of the health disparity over the life cycle have enlargement, convergence, or piecewise characteristics, the convergence model is used to test the evolution trend of the health disparity in this study, wherein the β convergence model capable of giving a statistical conclusion has been extensively applied to convergence research [30,31]. Then, the piecewise regression model is used to test whether a stage characteristic exists. The variables, symbols, and assignments used in the model are described in Table 2.

First, a health foundation model is built. We assume that the health index *HI* of the elderly *i* is the function of *age*. Screening is conducted based on goodness of fit, the Akaike information criterion (AIC) and the Schwarz criterion (SC) to obtain the quadratic function model reflecting the elderly health change trajectory, in which the higher the fit, the smaller the AIC and SC value, the better the model.

(1) $$HI_{i}=b_{0}+b_{1}age+b_{2}age^{2}+bX+\mu_{i}$$

wherein *b*_0_ is the initial health level; *b*_1_ signifies the degree of health varying with age, i.e., health loss rate; *b*_2_ signifies the nonlinear part of health changes; *µ_i_* is the stochastic part.

Next, dummy variables *p*_2_ and *p*_3_ are added to Equation (1) to represent observation periods to serve as Equation (2), where *p*_1_ is the base period, i.e., the observation year of 2011. Equation (2) is used to test the absolute income effect, and the absolute income effects of different groups are compared to explore the law of marginal utility of income. The two tests correspond to the first research content conducted in this study.

(2) $$HI_{i}=b_{0}+b_{1}age+b_{2}age^{2}+\alpha_{1}p_{2}+bX+\mu_{i}$$

When the sample year is 2014, Equation (2) can be expressed as

$$HI_{i}=\left(b_{0}+\alpha_{1}\right)+b_{1}age+b_{2}age^{2}+bX+\mu_{i}$$

The absolute income effect can be determined by the plus-minus sign and significance of α_1_ and α_2_. The smaller the health index value is, the better the health status will be. Thus, if the coefficients are significantly negative, it shows that the health level is improved with the increase of income, and the absolute income effect is positive. On the contrary, if the coefficients are significantly positive, it shows that the health level is not improved with the increase of income, and contrarily, the incidental effect of income increase exceeds the income effect. If the coefficients are not significant, it shows that the income level has little influence on health. The marginal health effect can be determined by the size and plus-minus sign of α_1_ and α_2_ in different income groups. For example, in the case the absolute values of α_1_ and α_2_ in the high-income group are smaller than those in the low-income group, it shows that compared to the low-income group, the health status of the high-income group is insensitive to income and the income effect has the law of diminishing marginal utility.

Equation (1) is added with grouping variables *g*_2_, *g*_3_, and *g*_4_ representing different income groups to serve as Equation (3), wherein, *g*_1_ is the base group, i.e., the lowest income group.

(3) $$HI_{i}=b_{0}+b_{1}age+b_{2}age^{2}+\delta_{1}g_{2}+\delta_{2}g_{3}+\delta_{3}g_{4}+bX+\mu_{i}$$

Similar to the derivation of Equation (2), the relative income effect can be determined by the significance and plus-minus sign of δ_1_, δ_2_, and δ_3_. If the coefficients are significantly negative, it shows that the high-income group has a significant health advantage compared to the low-income group. The changes in disparities among different groups can be determined by comparing the absolute values of δ_1_ − b_0_, δ_2_ − δ_1_, and δ_3_ − δ_2_, respectively. For example, in the case |δ_1_ − b_0_|>|δ_3_ − δ_2_|, it shows that the health disparity between the low-income group and the low- and middle-income group is larger than that between the middle- and high-income group and the high-income group. Apparently, we can re-explore whether the law of diminishing marginal utility of income exists using this test. Meanwhile, the values of relative income effects δ_1_, δ_2_, and δ_3_ in different periods can be compared to test the relations between the degree of income inequality and the health disparity, because the degree of inequality varies with different periods (see the Gini coefficient series in Table 2). This test part corresponds to the second research content.

Lastly, regarding the age pattern of income health effects, the β convergence model (i.e., Equation (4)) is used to test the evolution trend of the health disparity. β convergence theory originated from the “Iron Law of Convergence” in the neo-classical theory of economic growth, i.e., the law of diminishing marginal returns. The convergence in this paper refers to whether the health differences in different groups will shrink with age. β convergence currently has been extensively applied in studies on convergence (see [30,32] for more details); then, the piecewise regression model is used to test whether there is a stage characteristic.

(4) $$\ln\left(\Delta HI_{age+1}\right)-\ln\left(\Delta HI_{age}\right)=\gamma +\beta \ln\left(\Delta HI_{age}\right)+u_{age}$$

wherein ∆*HI* refers to the health disparity among groups; ln(∆*HI_age+_*_1_) − ln(∆*HI*_age_) signifies the logarithm of health disparity change rate between different groups with age; *u_age_* signifies the stochastic disturbance term; *β* is the convergence coefficient to show the convergence trend. In the case *β* < 0, the health disparity among different income groups gradually shrinks with the increase of age, which supports the convergence hypothesis. In the case *β* > 0, the health advantage of income through the accumulation over the life cycle causes the disparity to continue to expand, which supports the cumulative advantage hypothesis.

To explore whether the health disparity has a piecewise characteristic with age, the piecewise regression model (Equation (5)) is built. The advantage of piecewise regression lies in the fact that it can not only test whether there is a disparity among different stages, but also make the most of samples to improve the test precision.

(5) $$\Delta HI_{i}=b_{0}+b_{1}age+b_{3}\left(age-age^{*}\right)D_{1}+\mu_{i}$$

wherein

(6) $$D_{1}=\{\begin{array}{cc} 0, & age_{min}\leq age<age^{*}\\ 1, & age^{*}\leq age\leq age_{max} \end{array},$$

wherein *age* * signifies the piecewise point. If the coefficient *b*_3_ is positive, it shows that the health disparity among different income groups enlarges in the late stage; if the coefficient *b*_3_ is negative, it shows that the disparity shrinks in the late stage.

## 4. Result and Discussion

### 4.1. Sample Description

Based on the above mentioned idea, sample sizes, health averages, and standard deviations in different observation periods are summarized as shown in Table 3, wherein the sample age ranges from 65 to 100 and the samples losing key information are eliminated. The health level is the average health points of different income groups. The lower the points are, the healthier the group will be. In general, the elderly health points are 2 to 3, i.e., the health status thereof is between being good and fair; the middle- and high-income group and the low- and middle-income group get relatively low points, so they are healthier than the other groups, while the low-income group and the high-income group are relatively poor in health. With regards to whether the disparity among groups is significant and the possible cause thereof, they will be further explored later in this study.

### 4.2. Absolute Income and Relative Income Effects

Based on the proposed Equations (2) and (3), and CLHLS data, we know that the health effects of absolute income and relative income are verified, corresponding to the above research issues 1 and 2. The test results are shown in Table 4.

The left part of Table 4 corresponds to the absolute income effect; the right part corresponds to the relative income effect. According to Table 4, the model rationality is analyzed first. On the whole, the variables *age* and *age*^2 in all models pass the 0.01 significance test, and they all pass the F test at the significance level of 1%; furthermore, the coefficient *age* is significantly positive, and the coefficient *age*^2 is significantly negative, which shows the elderly health status declines with the increase of age, but the decline speed decreases progressively. The result conforms to the theoretical and realistic expectations and shows that the quadratic function as the health foundation model is rational; in addition, the influence of the control variable is not the research focus herein, thus, it is not discussed here. Then, the absolute income effect test is analyzed. Corresponding to columns 2–5 in Table 4, it is observed that *p*_2_ and *p*_3_ of all groups fail to pass the 0.05 significance test, which shows that the increase of income has an insignificant influence on health. It should be noted that coefficients *p*_2_ and *p*_3_ are both positive and health declines with the increase of income. In other words, the incidental effect of income increase surpasses the positive effect from the absolute income. Although the coefficients fail to pass the significance test, the health variation trend remains noteworthy. Comparing the coefficients *p*_2_ and *p*_3_ of different income groups, we found that the Low-income group and the Low- and middle-income group have a relatively large variation, and the Middle- and High-income group and the High-income group have a relatively small variation, which shows that health is less sensitive to income at a higher income level. This conclusion does not reject the diminishing marginal health effect of income. Finally, the relative income effect is analyzed. Corresponding to columns 6–8 in Table 4, it is observed that *g*_2_, *g*_3_ and *g*_4_ in all periods are significantly negative. In other words, compared to the lowest income group, the health status of other income groups is significantly improved. Further exploring the improvement range of different groups, we can see that, basically, the higher the ranking of income is, the better the health status will be. However, it is noteworthy that compared to the middle- and high-income group, the high-income group does not have the absolute advantage. The possible reason is not the research focus herein, but it still suggests that the income health effect may be in an inverted U-shape. When income reaches a certain level, its health effect begins to decline. The middle- and high-income group is possibly the effect inflection point in this study.

### 4.3. Age Characteristics of Health Returns of Income

Table 5 shows the test results of age characteristics of health returns of income. It should be noted that this part combines the low- and middle-income group and the middle- and high-income group into a middle-income group for discussion, i.e., discussing the health disparity among low-, middle-, and high-income groups. With regard to the reasons for the combination, first, in the test process, it is found that the low- and middle-income group and the middle- and high-income group have a relatively small health disparity, which can be roughly seen from the aforesaid descriptive statistics; second, it is aimed at ensuring a large disparity among groups and a small disparity within the same group, with no redundancy. This test corresponds to the abovementioned research issue 3.

As seen in Table 5, a convergence analysis on the health disparity is carried out. As in the upper part of Table 5, all convergence coefficients β are negative, which shows that the health disparity of different income groups shrinks with age. In other words, the cumulative effect hypothesis is not supported. Then, the significance of β is utilized to determine whether the health disparity is convergent. The result suggests that the significance of β in different income groups is inconsistent, and a general conclusion on whether the health disparity is convergent cannot be drawn. In general, the health disparity between the low-income group and the middle-income group is significantly convergent, but the health disparity between the high-income group and the middle-income group fails to pass the significance test. With regard to the possible reason for the difference in convergence, on the one hand, the low-income group often maintains a relatively optimistic attitude towards their health status assessment, which makes up for their disparity in objective health status to some extent, enabling the health disparity between the low-income group and the middle-income group to show a narrowing trend; on the other hand, the low- and middle-income group may have a higher health expectation, and have more access to their health information, thus, unlike the low-income group, they are not so optimistic towards self-rated health.

Then, we can observe whether the health return has a piecewise characteristic. For the piecewise test, the piecewise point should be determined first. In the absence of explicit theoretical support for the piecewise point, the graphic method is often adopted. In this study, the trend chart of the health disparity varying with age is observed, and it is found that the piecewise point is not significant (stability tests will be conducted for this judgment later in the study). However, compared to other points, slight changes can still be seen at the age of around 80. Thus, we choose the age of 80 as the piecewise point, for the purpose of drawing on experience and keeping consistent with existing literature [33]. As the test result shows in the lower part of Table 5, the model coefficients of the low- and middle-income group and the middle- and high-income group both fail to pass the significance test. The values of R^2^ and F are relatively low, and the overall model fails to pass the test, which shows that the setting of the linear model is irrational, age does not have a strong linear relation with the health disparity, and there is no piecewise characteristic from big to small, or small to big.

### 4.4. Further Discussion

In order to ensure the robustness of the above conclusions, this paper carries out a variable endogeneity test and stationarity test of health difference series. There are two main reasons for the endogeneity in the absolute income effect and relative income effect models. One is reverse causality, that is, while income affects health, health has a reverse social selection mechanism that affects income [34]. The other is omitted variables. There are many complicated individual characteristic factors influencing health. For example, personality, genes, and other variables that can influence both income and health are often omitted or unquantifiable [35], which may result in a deviation of research results. The endogenous problem is often solved by use of an instrumental variable or addition of a lagged term. In this study, the absolute income effect test model is aimed at exploring health changes with the year of 2011 as the base group. The effect is equivalent to the addition of lagged health status as an instrumental variable, which can not only eliminate omitted variables, but also avoid the correlation between explanatory variables, thereby solving the endogenous problem. The relative income effect is different from the absolute income effect. In this study, the cross-section data are used. Reverse causality and omitted variables are likely to occur. To control the endogeneity thereof, the two-way ANOVA model is used in this study. Observation periods and income groups serve as two factors influencing the health level. The health disparity among different income groups is explored by controlling the observation period variable. In essence, it is still equivalent to the addition of instrumental variables of lagged health level. The test result of the ANOVA is shown in Table 6. The overall model passes the 0.1 significance test, and the adjusted goodness of fit is 0.55. The low goodness of fit reflects the existence of other explanatory factors. However, here it is only emphasized that the observation period variable (lagged period variable) is insignificant, and the income groups pass the 0.05 significance test, which shows that even if endogeneity exists, it will not affect the above conclusion.

Then, the stationarity test refers to whether the health disparity tends to expand, converge, or remain unchanged with age. In this study, the ADF test is utilized to verify the rationality of the above hypothesis and conclusion. As shown in Table 7, the ADF test results show that the null hypothesis is rejected at the 5% significance level, that is, there is no unit root, and the difference in health at different ages is a stationary series. The conclusion is consistent with the above assumption and the research conclusion made by Link et al. [27] and supports the previous hypothesis.

## 5. Conclusions

Under the basic consensus that income is still an important factor influencing health, in the context of promoting the outline of the “Healthy China 2030 Plan”, exploring the relations between income and health, identifying important explanatory paths, and seeking the entry point of the health intervention policy is of great significance. In fact, the influence of income on health is manifested as the superposition of absolute income and relative income effects. Only by decomposing the two effects and comparing their relative importance in the same framework can we provide a scientific basis and empirical evidence for the top-level design of the health policy. In this study, based on CLHLS (2011, 2014, 2017) data, a research idea of bringing absolute income and relative income effects into the same framework is designed and plurality of analysis models are built to empirically test the paths and characteristics of the influence of income on health. Research shows that: (1) The positive effect of absolute income on health has not appeared, and the development of China’s macroeconomy and the increase in the level of national income have not led to a simultaneous increase in the health of the elderly. Meanwhile, the diminishing marginal health effect of income is not rejected in this study. In other words, compared to the middle- and high-income group and the high-income group, the health status of the low-income group and the low- and middle-income group is more sensitive to income. Thus, giving income subsidies to the low-income group and the low- and middle-income group is relatively effective to the overall improvement of the health level. (2) The relative income effect is significant, which means relative income is the main explanatory path influencing health, and there is a significant health disparity among different income groups. On the whole, higher income brings better health. However, it should be noted that the health status of the high-income group is not better than that of the middle-high income group; the health effect of income may have an inflection point, and the trend of exchanging income for health exists at a high income level. (3) As for the changes in health differences with age, the cumulative advantage hypothesis regarding health returns of income is not supported in this study, and the health disparity among different income groups is not enlarged with the increase of age. A general conclusion on whether the health disparity is convergent cannot be drawn in this study. There is no characteristic from big to small or small to big regarding the health disparity among different income groups. Actually, the results of the ADF test show that the health disparity varying with age is a random process and does not form a consistent trend. The stability tests in this study support the stability of the above model assumptions and conclusions.

The above research results provide a Chinese answer to the relations between income and health, which not only enrich and supplement related research, but also have important policy implications and play an enlightening role in exploring the countermeasures of improving health levels from the perspective of income. Specifically speaking: (1) The conclusions of this study show that there are no signs of the positive health effect of absolute income. This may be due to the improvement of self-health requirements arising from the changes in the macro environment, hierarchy of needs, etc., but is more likely to be because of weakening due to the incidental effect of income increase. The environmental health poverty brought by macroeconomic development, the increase of working time and work stress arising from income growth and so forth restrict the improvement of individual health status. Therefore, China ought to pay attention to the health costs arising from income growth while marching towards high income. The health policy is not only focused on the allocation of public medical and health resources, but also based on human development. We should be on the alert for such misunderstandings as “Income Uppermost” and “Exchanging Health for Income”. (2) Relative income is the main explanatory path influencing health, which shows that China’s elderly health level depends on the ranking of individuals in income sequence; the relative deprivation effect arising from income inequality does exist but weakens the health return brought by the increase of income level. Thus, the development of the health intervention policy may start with reducing income disparities, inequality in acquisition and utilization of critical resources, and other entry points; the development or reform of a public policy may be focused on optimizing medical resource allocation, improving the integrated level of basic medical services, elevating the equalization of the utilization rate of medical services, especially increasing subsidies and pensions for the low-income group, and enhancing the sense of security concerning elderly support to weaken the adverse impact of psychological risk factors on health. (3) The health return of income does not show the cumulative advantage characteristic, and the health disparity among different income groups is not enlarged with the increase of age, but a general conclusion that the disparity continues to shrink until it is convergent cannot be drawn. The health disparity between the low-income group and the middle-income group will gradually shrink, but the disparity between the middle-income group and the high-income group will persist throughout the life cycle, which shows that even in the old-age stage, the influence of external socioeconomic factors on health still transcends that of individual biological factors. The persistent existence phenomenon of the health gradient arising from the income gradient further demonstrates the importance of awareness and a timely intervention.

Finally, this study is also faced with several limitations: (1) Due to limited space and the boundary of research issues, in this study, the cohort effect is not considered in the exploration process of the absolute income effect. Thus, the age pattern of relations between income and health described herein includes not only the age effect, but also the cohort effect. The decomposition of age, period, and cohort effects will be the future research direction hereof. (2) For a possible endogenous problem, the ANOVA method is adopted herein to test the above conclusion, but specifically speaking, both methods are still essentially a correlation analysis on relations between income and health. In other words, they cannot distinguish the causal direction of relations between income and health status. Thus, it can be regarded as the starting point of a more extensive research plan herein. In the future, instrumental variables or other recognition strategies will be used to carry out a further cause-effect analysis so as to find some interesting phenomena and problems. (3) Tracking survey data are used herein, so the problem of sample selectivity bias may occur. In general, people at a high health risk are liable to dying or being lost for follow-up, and the living people among the samples may have a better health level. Thus, the elderly health level may be overrated in this study.

## Figures and Tables

**Table 1:** Quartile point of per capita income in each period.

Period (1)	Low (2)	Low-Middle (3)	Middle-High (4)	High (5)	Gini Coefficient
2008	≤678	678–1916	1916–4439	>4439	0.491
2011	≤1725	1725–4180	4180–10,677	>10,677	0.477
2014	≤2295	2295–5738	5738–12,237	>12,237	0.469

Note: Income in each period has been deflated according to the inflation index; the Gini coefficient data comes from the National Bureau of Statistics of China.

**Table 2:** Variable symbols and description.

Core Variables			
Symbols	Variables	Description	
*HI*	Self-rated health	“very good” = 1, “good” = 2, “fair” = 3, “bad” = 4, “very bad” = 5	
*age*	age	Range 65 to 100	
*p*_1_	Period when 2011	While period = 2011, *p*_1_ = 1, else, *p*_1_ = 0	
*p*_2_	Period when 2014	While period = 2014, *p*_2_ = 1, else, *p*_2_ = 0	
*p*_3_	Period when 2017	While period = 2017, *p*_3_ = 1, else, *p*_3_ = 0	
*g*_1_	Low income group	While group = Low, *p*_1_ = 1, else, *p*_1_ = 0	
*g*_2_	Low- middle group	While group = Low-middle, *g*_2_ = 1, else, *g*_2_ = 0	
*g*_3_	Middle-high group	While group = Middle-high, *g*_3_ = 1, else, *g*_3_ = 0	
*g*_4_	High group	While group = High, *g*_4_ = 1, else, *g*_4_ = 0	
Control variables **X**			
Variables	Description	Variables	Description
*sex*	While *sex* = ”woman”, *sex* = 1, else sex = 0	*drink*	While “yes” = 1, “else” = 0
*town*	While “yes” = 1, “else” = 0	*pension*	While “yes” = 1, “else” = 0
*smoke*	While “yes” = 1, “else” = 0	*childhood*	While “yes” = 1, “else” = 0

**Table 3:** Sample data description.

Periods	Sample Size	All Average Level (Deviation)	Low-Group Average Level (Deviation)	Low-Middle Average Level (Deviation)	Middle-High Average Level (Deviation)	High Average Level (Deviation)
2011	8944	2.17 (0.63)	3.13 (0.74)	1.52 (0.64)	1.58 (0.69)	2.21 (0.74)
2014	5424	2.71 (0.54)	3.04 (0.61)	2.54 (0.74)	2.32 (0.72)	2.30 (0.76)
2017	4244	2.48 (0.51)	2.92 (0.57)	2.67 (0.66)	2.42 (0.69)	2.44 (0.70)

**Table 4:** Test of absolute income and relative income effects.

Variables	Absolute Income Effects				Relative Income Effects		
	Low	Low-Middle	Middle-High	High	2011	2014	2017
*age*	0.102 ***	0.095 ***	0.103 ***	0.100 **	0.081 ***	0.123 ***	0.098 ***
*age*^2	−0.001 ***	−0.001 ***	−0.001 ***	−0.001 ***	−0.001 ***	−0.001 ***	−0.001 ***
*p*_2_	0.072	0.110	0.060	0.040	−	−	−
*p*_3_	0.065 *	0.095	0.052 *	0.054 *	−	−	−
*g*_2_	−	−	−	−	−0.181 ***	−0.173 ***	−0.151 ***
*g*_3_	−	−	−	−	−0.266 ***	−0.244 ***	−0.217 ***
*g*_4_	−	−	−	−	−0.252 ***	−0.235 ***	−0.213 ***
**X**	−	−	−	−	−	−	−
R^2^	0.483	0.431	0.518	0.595	0.5543	0.626	0.664
F	13.764	10.096	15.705	46.339	41.342	56.453	68.745
*p*	0.000	0.000	0.000	0.000	0.000	0.000	0.000

Note: R^2^ and F mean fitting degree and F-test value; “−“means that corresponding variables are not included in the model; ***, **, and * respectively indicate that the regression coefficient passes the significance test at the significance levels of 1%, 5%, and 10%. The setting of the control variable **X** is aimed at ensuring the model rationality, which is slightly omitted for indexing, because it is not the research focus herein.

**Table 5:** Health difference characteristics.

Convergence Analysis						
Variables	2011		2014		2017	
	Low-Middle	Middle-High	Low-Middle	Middle-High	Low-Middle	Middle-High
β	−0.971 **	−0.841 *	−0.937 ***	−0.732	−0.547 **	−0.610
R^2^	0.347	0.412	0.338	0.458	0.316	0.349
F	28.748	36.211	28.283	43.472	23.459	35.398
*p*	0.000	0.000	0.000	0.000	0.000	0.000
**Piecewise Characteristic**						
Variables	2011		2014		2017	
	Low-Middle	Middle-High	Low-Middle	Middle-High	Low-Middle	Middle-High
*age*	0.002	0.004	0.006	0.007	0.008	0.004
*Age − age*	0.003	0.001	0.009	0.009	0.003	0.008
R^2^	0.157	0.149	0.126	0.131	0.198	0.121
F	4.723	3.244	2.539	2.638	6.995	1.651
*p*	0.065	0.081	0.096	0.088	0.012	0.129

Note: β means convergence coefficient; R^2^ and F mean fitting degree and F-test value; ***, ** and * respectively indicate that the regression coefficient passes the significance test at the significance levels of 1%, 5%, and 10%.

**Table 6:** Stability test.

Variables	Sum Square	Degree of Freedom	Mean Square	F	Significance
Calibration	4.28	5	0.86	4.26	0.07 *
intercept	79.52	1	79.52	395.60	0.00
group	2.98	3	0.99	4.95	0.04 **
period	1.29	2	0.65	3.21	0.11
error	1.21	6	0.20		
totle	85.00	12			
R^2^ = 0.754, ${\bar{R}}^{2}=0.549$					

Note: F means F-test value; **, and * respectively indicate that the regression coefficient passes the significance test at the significance levels of 5% and 10%.

**Table 7:** Health disparity stability.

Periods	2011		2014		2017	
Groups	Low-Middle	Middle-High	Low-Middle	Middle-High	Low-Middle	Middle-High
stationarity	−3.96 **	−5.93 ***	−5.23 ***	−6.94 ***	−4.49 ***	−7.78 ***

Note: *** and ** respectively indicate that the regression coefficient passes the significance test at the significance levels of 1% and 5%.

## Data Availability

The study did not report any data.
